# AI-Assisted Carotid Ultrasound for Nonexpert Use in Primary Care Cardiovascular Prevention: User-Centered Iterative Development Study

**DOI:** 10.2196/105250

**Published:** 2026-10-08

**Authors:** Anna Sjöström, Christer Grönlund, Karolina Jonzén, Patrik Wennberg, Åsa Hörnsten, Thorbjörn Lundberg, Anni-Maria Pulkki-Brännström, Luisa Ebeling, Albin Dahlin Almevall

**Affiliations:** 1 Department of Nursing Umeå University Umea, Västerbotten Sweden; 2 Department of Diagnostics and Intervention Umeå University Umea, Västerbotten Sweden; 3 Västerbotten County Umea, Västerbotten Sweden; 4 Department of Public Health and Clinical Medicine Umeå University Umea, Västerbotten Sweden; 5 Department of Epidemiology and Global Health Umeå University Umea, Västerbotten Sweden; 6 Umeå University Umea, Västerbotten Sweden; 7 Department of Public health and Clinical Medicine Umeå University Umea, Västerbotten Sweden; 8 Department of Health and Medical Care Region Norrbotten Luleå, Norrbotten Sweden

**Keywords:** AI, artificial intelligence, atherosclerosis visualization, cardiovascular disease prevention, carotid ultrasound, implementation, nonexpert operators, primary care, risk communication, task shifting, user-centered design

## Abstract

**Background:**

Carotid ultrasound is traditionally confined to specialist settings because examination quality and diagnostic reliability are highly dependent on the operator’s technical skill and experience. Recent advances in AI-assisted ultrasound may enable task shifting to primary care staff and support the integration of personalized visual risk communication into routine preventive care. Visualization of subclinical atherosclerosis has been shown to strengthen cardiovascular risk communication and support preventive engagement. However, limited evidence exists on how such technology can be practically integrated into routine primary care workflows and what conditions are required to support use by nonexpert operators.

**Objective:**

This study aimed to describe an iterative user-centered design process enabling nonexpert use of AI-assisted carotid ultrasound for visualizing subclinical atherosclerosis in primary care cardiovascular disease prevention.

**Methods:**

We conducted a user-centered, iterative study within the Västerbotten Intervention Programme in northern Sweden. Fourteen nurse assistants from 4 primary care centers representing urban, rural, and remote rural settings tested an AI-assisted carotid ultrasound prototype with real-time guidance and artery segmentation in their primary care work environment, while 15 nurse assistants participated in semistructured interviews. Structured system-interaction observations were conducted during prototype testing. Interview data were analyzed using inductive thematic analysis informed by Braun and Clarke’s approach, while data from structured system-interaction observations were analyzed pragmatically to identify usability and workflow issues relevant to the iterative development process.

**Results:**

The thematic analysis of interview data identified 4 themes describing nurse assistants’ experiences and conditions for implementation: technology that fits the everyday primary care work; system guidance as a prerequisite for effective use; practical, peer-supported learning for confidence in clinical use; and confidence in the patient encounter. Observations identified recurring usability challenges related to ergonomics, probe handling, interface navigation, image acquisition, and time pressure. These findings led to iterative refinements, including redesigned interface navigation, simplified data entry, added audio feedback, enhanced probe support, and revised training materials.

**Conclusions:**

This user-centered study identified key practical, organizational, and educational conditions for integrating AI-assisted carotid ultrasound into routine preventive care by nonexpert staff in primary care. The findings suggest that successful implementation will depend not only on technical capability but also on ergonomic and workflow fit, intuitive system guidance, peer-supported learning, and confidence in patient communication. These findings provide a foundation for future validation and implementation studies.

## Introduction

### AI-Assisted Carotid Ultrasound and Operator Dependence

Carotid ultrasound is highly operator-dependent, with quality and diagnostic reliability relying on user expertise [1,2]. In Sweden, examinations are typically performed in hospital settings by highly trained biomedical scientists, which has limited broader use outside specialized care. Against this background, recent advances in AI have opened new possibilities for improving standardization and reducing variability in ultrasound imaging [3]. AI-assisted systems can provide automated guidance and real-time image analysis during scanning, with the potential to support clinically relevant examinations by nonexpert users [2,4]. In this context, such technologies may facilitate task shifting within primary care and support integration into routine preventive consultations, although successful implementation depends on adequate training, organizational support, and user confidence [5,6].

Previous research has shown that automated carotid intima-media thickness (cIMT) measurements are reproducible even when performed by novice operators [7], suggesting that vascular ultrasound assessments may be amenable to task shifting when supported by appropriate technology. Semiautomated systems for cIMT assessment are also already commercially available.

In contrast, manual detection of carotid plaques is associated with substantial interobserver variability, particularly for smaller plaques [8], highlighting the need for supportive tools to improve consistency in clinical practice. Although recent studies have proposed methods for automated plaque detection and segmentation [9,10], such tools remain less established than those for cIMT assessment. Furthermore, there is limited evidence regarding how these technologies can be integrated into routine clinical workflows and used by nonexpert operators. However, recent evidence suggests that AI-assisted carotid ultrasound may improve the performance of nonexpert operators. In a prospective controlled trial, an intelligent handheld ultrasound system improved diagnostic accuracy, agreement with specialist assessments, and examination efficiency among nonexpert general practitioners performing carotid examinations in community settings [11]. Taken together, these developments suggest that AI-assisted carotid ultrasound may support broader use in cardiovascular disease (CVD) prevention by improving access to clinically relevant visualization.

### CVD Prevention and Risk Communication

From a preventive perspective, CVD remains a leading cause of morbidity and mortality worldwide, and the burden is projected to rise substantially as populations age [12,13]. In addition, beyond the personal impact on individuals and families, CVD places a substantial strain on health care systems and society, highlighting the importance of effective preventive strategies [14]. Primary prevention through lifestyle modification, including a healthy diet, physical activity, smoking cessation, and weight management, can substantially reduce cardiovascular risk [15,16], and adherence to preventive medication is associated with reduced cardiovascular mortality [17]. Despite this, sustaining healthy lifestyle behaviors and maintaining adherence to preventive medication remain challenging for many individuals [18-20].

In this context, effective communication plays an important role in supporting patients’ understanding, motivation, and adherence to preventive strategies [21]. Traditional cardiovascular risk communication based on numerical risk scores, such as Systematic Coronary Risk Evaluation 2 (SCORE2), which is widely used in European primary care, is often perceived as abstract and difficult for patients to personally relate to [22]. Consequently, increasing attention has been directed toward communication approaches that strengthen patients’ understanding of their individual risk and support sustained motivation for lifestyle change [23,24]. One such approach involves the visualization of subclinical atherosclerosis, which provides a more concrete and personally relevant representation of cardiovascular risk.

### Visualization of Subclinical Atherosclerosis

The visualization of subclinical atherosclerosis provides a concrete and personally relevant way to illustrate early vascular disease processes. In this context, communicating visible signs of underlying atherosclerosis may enhance engagement in preventive behaviors more effectively than abstract statistical estimates alone [25]. Carotid plaque and cIMT are well-established predictors of future cardiovascular events [26]. Evidence supporting this approach comes, for example, from the VIPVIZA (Västerbotten Intervention Programme–Visualization of Asymptomatic Atherosclerotic Disease for Optimum Cardiovascular Prevention) randomized controlled trial. In VIPVIZA, personalized pictorial feedback showing vascular age and plaque burden, combined with lifestyle counseling, led to improved health behaviors and more favorable cardiovascular risk factor trajectories, including increased initiation of lipid-lowering therapy [27-29]. These effects were reflected in a slower progression of cIMT after 3 years [30,31]. More recently, the 6-year VIPVIZA follow-up provided further evidence of beneficial effects on cardiovascular risk factors, including substantial reductions in low-density lipoprotein cholesterol [32]. In addition, participants also reported that the visualization increased their understanding of cardiovascular risk and motivation to adopt healthier behaviors [33]. However, in VIPVIZA, the ultrasound examinations were performed by experts at a research center outside routine primary care, and visual feedback was delivered later by mail and discussed with participants in a follow-up telephone call [30]. Taken together, this highlights a gap between the demonstrated benefits of visualization and its integration into routine primary care practice.

### Primary Care Context and Implementation Potential

To address this gap, integrating visualization directly into preventive health care visits could strengthen the communicative potential of this approach by enabling immediate dialogue and interpretation between patients and clinicians. In this regard, the Västerbotten Intervention Programme (VIP) provides a relevant infrastructure for exploring such integration. VIP is a population-based cardiovascular prevention program embedded in primary care in northern Sweden. Residents aged 40 years, 50 years, and 60 years are invited to their local primary care center, where nurse assistants conduct clinical assessments, including blood sampling and blood pressure measurements, before a preventive health dialogue with a nurse [34]. The program has demonstrated positive long-term effects on population health [35], and its established workflows and continuous professional training provide favorable conditions for integrating new preventive methods.

Furthermore, in geographically large and sparsely populated regions such as northern Sweden, access to specialized diagnostic services is often limited by long travel distances and the centralization of expertise in hospital settings. Strengthening preventive capacity within primary care is therefore an important strategy for promoting health equity in rural and remote areas, aligning with Sweden’s ongoing health care reforms that emphasize accessible, coordinated, and locally delivered care [36,37]. This broader context further underscores the need for approaches that enable accessible, nonspecialist use of imaging technologies within primary care.

### Study Rationale and Objective

The present study was conducted within the PRIMVIZA (Primary Care Prevention of Cardiovascular Disease Using Visualization of Subclinical Atherosclerosis for Improved Risk Communication) project, which builds on the VIP infrastructure and the experience gained from VIPVIZA as a translational step to explore how AI-assisted carotid ultrasound could be integrated into routine primary care prevention [38]. While previous research has demonstrated the potential of visualizing subclinical atherosclerosis to support cardiovascular risk communication, the implementation of such approaches within everyday primary care remains limited.

In this context, PRIMVIZA aims to develop a workflow in which nurse assistants perform an AI-assisted carotid ultrasound examination during the VIP visit, with the resulting visualization used in the subsequent nurse-led health dialogue to support communication about cardiovascular risk and motivate preventive lifestyle changes. To ensure that the emerging system and workflow are compatible with everyday clinical practice, the project applies principles of user-centered design, emphasizing early and continuous involvement of end users to improve usability, acceptability, and contextual relevance [39,40].

However, it remains unclear how such an approach can be integrated into existing primary care workflows and what conditions are required for nonexpert operators to use AI-assisted carotid ultrasound in routine preventive practice. Therefore, the aim of this study was to describe an iterative user-centered design process enabling nonexpert use of AI-assisted carotid ultrasound for visualizing subclinical atherosclerosis in primary care cardiovascular disease prevention.

## Methods

### Study Context

The present study was conducted within the VIP, which is a long-standing population-based cardiovascular prevention program integrated into primary care in northern Sweden [34]. Residents aged 40 years, 50 years, and 60 years in Västerbotten County are invited to their local primary care center, where nurse assistants perform clinical assessments, including venous blood sampling for lipid and glucose analysis and blood pressure measurements. Before the visit, participants complete a questionnaire covering lifestyle and psychosocial factors. The results are summarized in a visual “star profile” that structures a personalized health dialogue with a nurse focusing on cardiovascular risk, lifestyle habits, and health in general. VIP includes continuous professional training to support preventive routines and communication practices.

Building on this preventive infrastructure, the VIPVIZA trial, on which the present PRIMVIZA project is based, introduced carotid ultrasound as an additional tool for risk communication by adding an examination performed by biomedical analysts at a research center, with visual feedback sent to participants by mail and followed by a telephone call from a nurse to discuss the results. In total, 3532 participants were recruited, and ultrasound examinations were performed at baseline and follow-ups at 3, 6, and 10 years (ongoing), with low attrition, generating substantial regional experience with vascular imaging for preventive communication [28,30,32].

The PRIMVIZA project aims to investigate and develop methods for a large-scale integration of an AI-assisted carotid ultrasound examination into the existing VIP sampling visit, with nurse assistants performing the scan as part of the health examination at the primary care center. The resulting real-time visualization is intended for use in the subsequent nurse-led health dialogue to support preventive communication and behavior change. PRIMVIZA is a multidisciplinary initiative involving researchers from medicine, nursing, medical engineering, health economics, and industrial design, together with primary care nurses, nurse assistants, and VIP participants. This collaborative structure supports the iterative development process and early evaluation of a workflow aligned with existing roles and routines in VIP.

### AI-Assisted Carotid Ultrasound System (Prototype)

The following description refers to the baseline prototype used during the initial testing cycles of the PRIMVIZA user-centered design process. The prototype combined a commercially available ultrasound system with custom-developed AI-assisted software (AI Carotid Scanner), which was developed specifically for this project and iteratively refined throughout the study.

The prototype consisted of three primary hardware components: (1) a touchscreen interface for graphical user interaction (24” Dell P2424HT), (2) a PC that ran the real-time AI model and associated software (Lenovo P16v), and (3) an ultrasound system (GE Vivid IQ Premium) with an L9 probe (GE Healthcare) for image acquisition. Ultrasound images were captured using a video capture device (Elgato Cam Link 4K) and integrated into the software for visualization and analysis. Ultrasound imaging was performed using the same preset settings as those used in the VIPVIZA study, from which the AI training images were derived, to maximize similarity between acquired images and the training data and thereby reduce out-of-domain shift in the AI model.

All modules were mounted on a wheeled stand (Figure 1). The complete prototype, including all mounted components, had approximate base dimensions of 59.5 × 54.5 cm (W × D). The system required access to a standard electrical outlet and sufficient space for the operator to be seated beside the patient. All processing was performed locally on the PC, and no internet connectivity was required.

**Figure 1:**
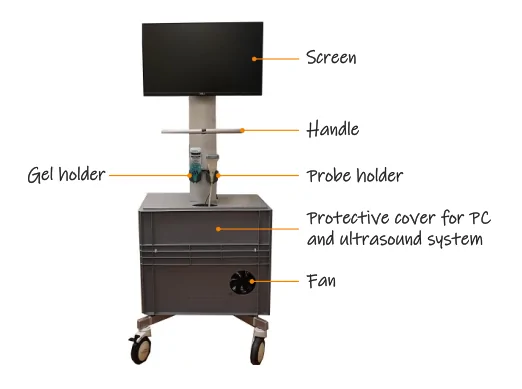
Baseline system prototype.

The AI Carotid Scanner software was initially conceptualized using Axure RP 8, a prototyping tool, to define the main layout and core functionality through rapid prototyping. During the examination, the integrated AI model, based on a convolutional neural network (YOLOv8), performed real-time segmentation of the carotid artery at approximately 10 images per second. The model was trained on approximately 3000 annotated carotid ultrasound images from the VIPVIZA study. A detailed technical evaluation of the model’s performance in segmenting the common carotid artery, carotid bulb, and internal and external carotid arteries has been reported elsewhere [41]. The segmentation output was visualized in the interface, with the artery outlined to assist the operator in locating and tracking the vessel (Figure 2). This real-time segmentation overlay provided visual guidance during scanning to support probe positioning and vessel tracking by nonexpert users. Additional technical specifications of the prototype system and AI model are provided in Multimedia Appendix 1.

**Figure 2:**
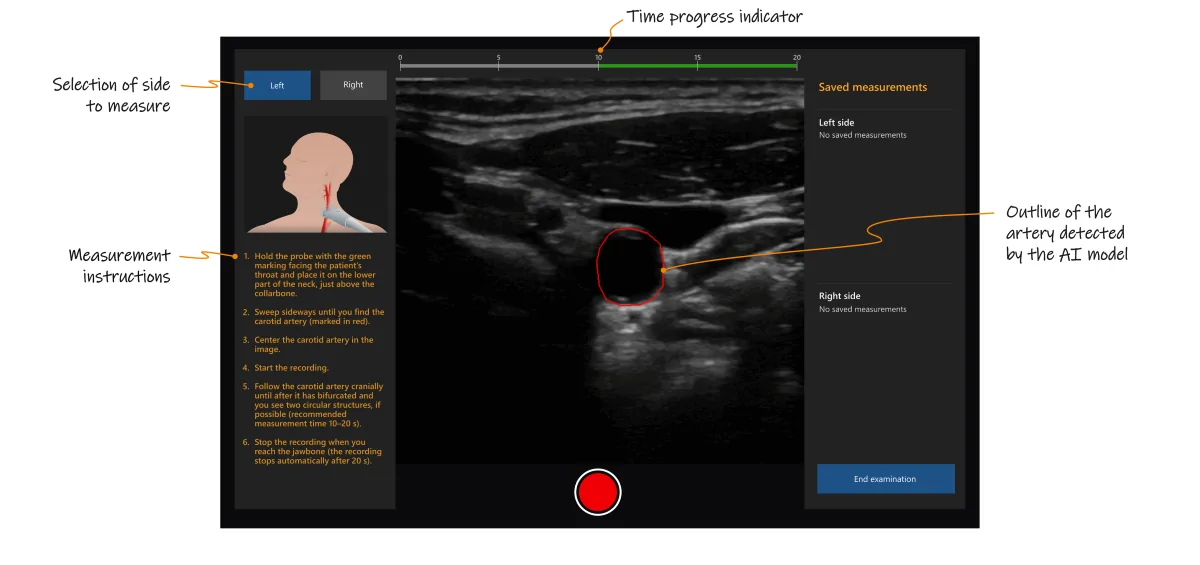
Graphical user interface of the baseline system prototype.

The examination procedure supported by the prototype was structured as follows. Before initiating a scan, the operator entered the patient’s age and sex in the graphical user interface (Figure 3). The software then guided the user through the scanning process, with the goal of acquiring images of sufficient quality for both carotid arteries. For each side, the probe was first placed just above the collarbone to locate the common carotid artery, where it appeared with an overlaid artery outline in the graphical user interface. At this starting position, the operator initiated the recording and moved the probe cranially along the vessel, past the carotid bifurcation and toward the mandibular angle, to capture the relevant arterial segment. Each recording sequence was limited to 20 seconds, requiring the operator to continuously follow the artery during this time window. After the recording, a result screen displayed the assessed image quality (approved or low) together with guiding tips for improving image acquisition if needed.

**Figure 3:**
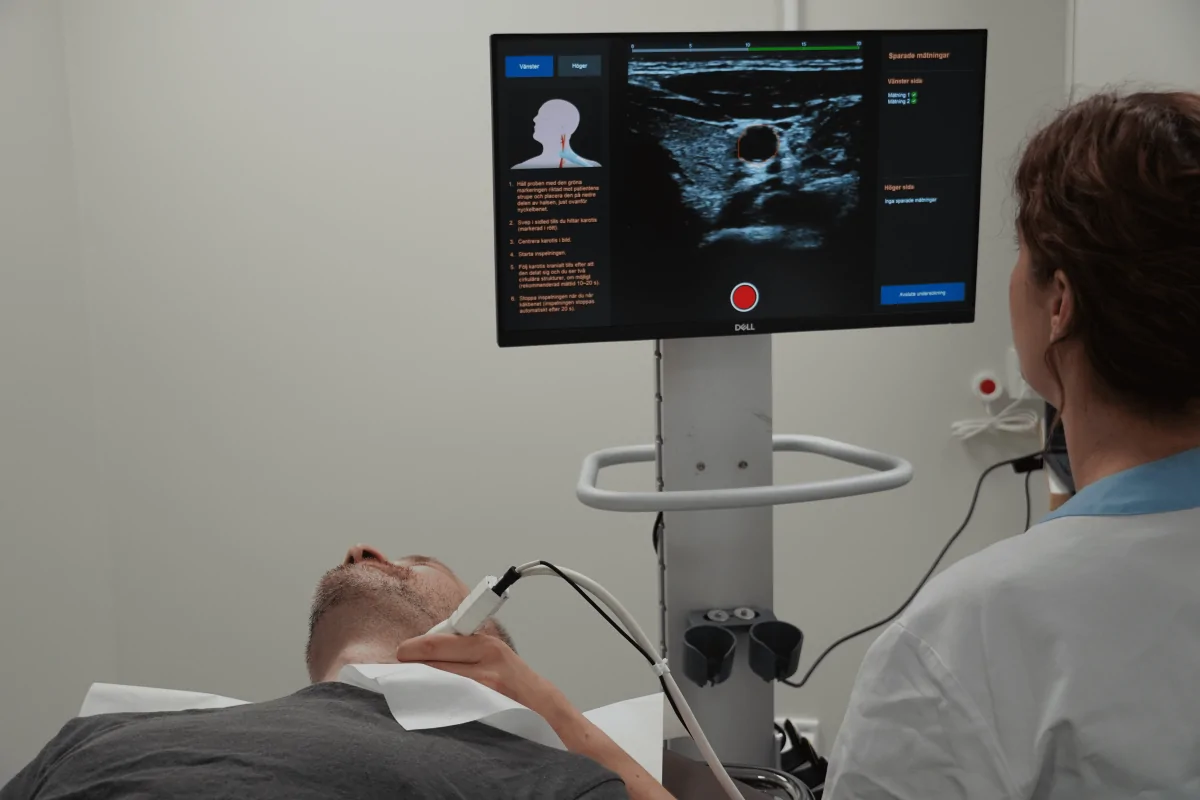
Overview of the examination setup showing the operator performing the scan on a sample patient, with the prototype system and screen positioned next to the examination bed.

The prototype was developed in-house at Umeå University Hospital by a multidisciplinary team including project managers, software developers, and AI specialists, together with experts in ultrasound imaging and clinical workflow. Prior to on-site evaluation in health care environments, the prototype underwent internal testing to identify and resolve design flaws and to assess safety considerations, ensuring readiness for user trials.

### Participants and Study Setting

The present study was conducted at 4 primary care centers, operated by Region Västerbotten (publicly funded health care), in Västerbotten County, Sweden, selected to represent organizational and geographical diversity, including 2 urban, 1 rural, and 1 remote rural setting. These centers serve populations ranging from approximately 3000 to 12,000 listed patients, reflecting variation in access and organizational context across the region. All centers routinely deliver preventive cardiovascular care through the VIP, making them a relevant real-world setting for evaluating the PRIMVIZA approach.

Recruitment occurred during the annual VIP training days, where the PRIMVIZA project was presented to nurse assistants and nurses from multiple primary care centers. Nurse assistants who expressed interest in learning more and participating in prototype testing registered their contact details for follow-up. Managers at these centers were subsequently approached with written and verbal information, and centers were included only after formal managerial approval. To ensure feasibility in later implementation stages, participation also required agreement from both VIP nurse assistants and VIP nurses at each center.

All participating nurse assistants were employed in primary care and experienced in conducting the clinical assessments that form part of the VIP health examination, including blood sampling and blood pressure measurements. Nurse assistants in Sweden have 3 years of formal upper-secondary health care education and routinely conduct basic clinical examinations in primary care. Participating nurse assistants had previous experience with bladder scanning to assess urinary retention, involving the use of handheld ultrasound devices, but none had previous experience with AI-assisted health care technologies. In total, 15 nurse assistants, all women, participated in the study; 14 participated in prototype testing and system interaction observations, while all 15 participated in interviews.

### User-Centered Design Approach and Iterative Process

The overall methodology followed a tailored, user-centered development process designed specifically for the PRIMVIZA context and objectives, informed by established principles of user-centered design described in previous research [39,40]. Nurse assistants, the intended end users, were actively involved throughout the iterative design and evaluation process conducted in their real clinical environment.

Each cycle included on-site prototype testing supported by parallel data collection by multiple members of the multidisciplinary research team, focusing on usability, workflow fit, and professional experiences. As shown in Figure 4, the iterative process consisted of repeated cycles of testing, evaluation, and redesign, where findings from each visit informed refinements to the system and associated training approach before the subsequent testing session. After each visit, findings were synthesized and translated into refinements of the training, hardware setup, software, and user interface. The updated version was then evaluated in the following cycle to ensure that changes were grounded in end user needs and real-world clinical conditions.

**Figure 4:**
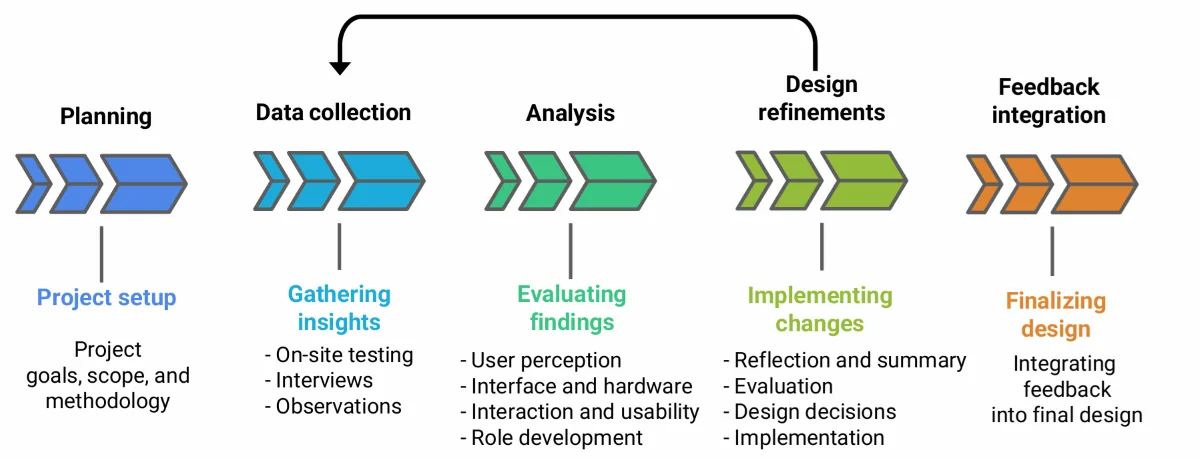
Iterative user-centered design and refinement process in PRIMVIZA (Primary Care Prevention of Cardiovascular Disease Using Visualization of Subclinical Atherosclerosis for Improved Risk Communication).

This process continued across participating primary care units until all recruited sites had been visited and the prototype design had stabilized, with later iterations mainly confirming usability improvements and identifying only minor refinements. For example, later iterations confirmed that earlier refinements to interface navigation and probe handling guidance resolved issues identified during the initial testing sessions.

### Data Collection

#### Procedure at Primary Care Centers

On-site data collection visits were conducted between February and November 2025, during which members of the research team visited each participating primary care center. The planned duration for each visit was approximately 2 hours to allow sufficient time for introductory instruction, demonstration, prototype testing, observation, and interviews. In line with the aims of the study, the introductory instruction was intentionally kept concise to explore how nurse assistants engaged with the system following brief instructions and hands-on testing in a realistic clinical setting. Due to staff illness at 2 centers, the available time was reduced to 1.5 hours, which required the research team to further shorten the introductory instruction. This resulted in testing conditions that closely reflected typical time constraints in routine primary care practice. At 1 center, 2 visits were conducted to enable participants to test scanning of both carotid arteries and to include a nurse assistant who had been absent during the first visit.

All visits followed the same overall structure. First, participating nurse assistants received a concise introductory instruction session about the project, including the purpose of the prototype, the scientific rationale for visualizing subclinical atherosclerosis, basic carotid anatomy, and key ultrasound imaging principles. The training content was developed in collaboration with biomedical scientists experienced in carotid ultrasound imaging to ensure alignment with current best practices and included a short practical demonstration of probe handling, scanning technique, and the AI-assisted interface workflow.

After the demonstration, 14 nurse assistants individually tested the prototype in the clinical environment while being observed by the research team, who completed structured observation forms focusing on usability and workflow fit. One additional nurse assistant attended the introductory instruction but did not perform the prototype examination and therefore contributed only to the interview data. A healthy 30-year-old male member of the medical engineering team, who had been involved in the development of the prototype, served as a standardized scanning participant during all prototype testing sessions. Using the same individual across sites and iterations ensured comparable anatomical conditions and reduced the risk of unexpected clinical findings during this formative phase. During testing, his role was limited to serving as the scanning participant. He did not provide instructions, guidance, or assistance to participants during the examinations. Finally, participants took part in semistructured interviews.

#### System Interaction Observations

Observations were conducted during prototype testing to examine nurse assistants’ interaction with the system in a realistic clinical environment. All observations were conducted by the same researcher (KJ). A structured, task-based observation protocol (Multimedia Appendix 2), developed specifically for this study, was used to cover key components of the examination procedure, including probe handling, navigation of the user interface, and responses to AI guidance cues. Participants were encouraged to think aloud while performing the tasks to facilitate identification of cognitive challenges and usability barriers. The observer documented task completion and recorded any deviations, uncertainties, or workarounds in the observation protocol.

The level of instructor support varied iteratively across sessions. At the first site, a fly-on-the-wall approach was used, where users performed the procedure without assistance to reveal areas where the prototype lacked clarity. As development progressed, brief clarifications and demonstrations were provided when needed to support participants in progressing through the tasks. All instructor interventions were documented in the same observation protocol.

#### Semistructured Interviews

Semistructured interviews were conducted with participating nurse assistants, using a study-specific interview guide (Multimedia Appendix 3), to explore their perspectives on the prototype, its usability, and its potential integration into existing VIP routines, as well as their views on training materials and the support, knowledge, and organizational conditions required for implementation in routine clinical practice. The interviews were conducted by 2 researchers. AS conducted 8 interviews and ADA conducted 2 interviews. Both interviewers were registered nurses with specialist training as primary care nurses, and neither had a prior relationship with the participants. A common semistructured interview guide was used across all interviews. Interview experiences and emerging issues were discussed within the research team during data collection, and both interviewers subsequently participated in the analysis and interpretation of the interview data.

Depending on practical conditions at each primary care center, interviews were performed either immediately after prototype testing or on the following day, and either on site or online. Across the 4 centers, this resulted in a combination of same-day individual interviews, next-day online interviews, and 1 online focus group interview, which was also conducted the following day. In total, 15 nurse assistants participated in 9 individual interviews and 1 focus group interview. One interview participant had attended the introductory instruction but had not personally tested the prototype and therefore contributed perspectives on implementation and the potential integration of ultrasound into VIP rather than experiences of prototype use. Interview data collection was concluded when all recruited primary care centers had completed the planned testing and interview procedures. Consistent with the iterative user-centered design of the study, the end point was determined by completion of the planned site-based testing cycles and stabilization of the prototype rather than by a predefined criterion of data saturation. Later iterations primarily confirmed previously identified usability needs and resulted in only minor refinements.

Interview duration ranged from 9 to 33 minutes for individual interviews (median 24, IQR 20-26 minutes), while the focus group interview lasted 41 minutes. The interviews were audio-recorded with participants’ consent, transcribed verbatim, and anonymized prior to analysis.

### Data Analysis

#### Overview

Given the formative and user-centered purpose of the study, the overall analytic approach was pragmatic and closely integrated with the iterative development process. Observation and interview data served complementary purposes and were analyzed separately.

#### Observation Data

Data from the structured system-interaction observations were analyzed pragmatically, focusing on identifying usability barriers and facilitators relevant to the iterative development process. After each test session, the research team reviewed completed observation protocols and field notes to determine which tasks were difficult, incomplete, or required additional support.

Observed challenges and facilitators were discussed within the research team to identify recurring patterns across participants and to assess their potential impact on task performance. Particular attention was given to issues that appeared consistently or were judged to significantly affect usability and workflow integration. For observation items that were systematically recorded across participants, descriptive participant-level counts were compiled to indicate how frequently specific usability issues were observed. These counts were used descriptively to illustrate the recurrence of observed issues and were not intended for statistical inference.

The analytic approach was informed by principles described by Medlock et al [42], emphasizing the early identification of usability problems and supporting timely modification when issues are evident and solutions are clear, rather than delaying action until confirmed through large sample sizes or formal statistical validation. As the observer (KJ) was also involved in prototype development, observation findings were discussed within the broader research team to incorporate perspectives from researchers not directly involved in conducting the observations.

#### Interview Data

Interview data were analyzed using inductive thematic analysis informed by Braun and Clarke [43]. The analysis drew on the phases of familiarization, coding, theme development, review, definition, and reporting, with themes developed as patterns of meaning across the dataset.

The first and last authors both coded the complete interview dataset inductively, without a predefined coding framework or codebook. They subsequently discussed their interpretations and developing patterns of meaning. These discussions were not undertaken to establish intercoder agreement or coding consensus, but to explore different interpretations and deepen the analysis. Codes and preliminary themes were subsequently discussed within the full research team and further developed and refined through an iterative process.

Mind maps were used to examine relationships between codes and to develop candidate themes. Throughout the analysis, decisions regarding coding and theme refinement were documented to maintain transparency and support confirmability. Field notes and a reflexive journal were used to contextualize interpretations and enhance analytic rigor. All transcripts were anonymized prior to analysis. The full thematic analysis was conducted after completion of all interviews and system-interaction observations and resulted in themes reflecting broader patterns of meaning in participants’ experiences of the technology and perceived conditions for its integration into primary care.

During data collection, however, concrete prototype-related feedback expressed by participants, such as comments on ergonomics, device design, and practical use, was documented separately and shared iteratively with the medical technology team to inform ongoing prototype development. This formative feedback process was distinct from the subsequent thematic analysis of the complete interview dataset.

#### Iterative Refinement Process

Findings from the structured system-interaction observations and formative prototype-related feedback identified during the interviews informed ongoing iterative refinements of the system. During data collection, concrete comments concerning the prototype were documented and communicated to the medical technology team. Following each test cycle, these formative insights were reviewed by the research and development team and used to guide targeted revisions to hardware, software, the user interface, training materials, and workflow. This iterative process allowed identified usability issues to be addressed progressively throughout the study. The full thematic analysis of the interview dataset was conducted after completion of data collection and was therefore not part of this iterative refinement process.

### Ethical Considerations

The study was conducted in accordance with the Declaration of Helsinki [44]. Ethical approval was obtained from the Swedish Ethical Review Authority (Dnr 2024-03932-01). All participants received written and verbal information about the study, including its purpose, procedures, voluntary nature, and the right to withdraw at any time without consequences. Participation in prototype testing and interviews was considered to constitute informed consent. The involvement of the medical engineering team member who served as the standardized scanning participant was covered by the same ethical approval, and he provided informed consent to participate in the prototype testing sessions.

To protect privacy and confidentiality, all interview recordings and transcripts were pseudonymized and stored securely in accordance with Umeå University data management procedures. Only members of the research team had access to identifiable data, and all findings are reported at the group level to prevent identification of individual participants or primary care centers.

Prior to study initiation, a risk analysis was conducted to identify potential ethical and organizational risks. Identified risks included breaches of confidentiality and time away from routine clinical duties. These risks were mitigated through secure data handling procedures and by obtaining approval from primary care center management before participation.

Primary care centers were compensated for staff time spent participating in the study. No financial compensation was provided directly to individual participants.

This study is reported in accordance with SRQR (Standards for Reporting Qualitative Research) [45]. The completed SRQR checklist is provided in Multimedia Appendix 4.

## Results

### Overview

The results are presented in 4 sections describing participant characteristics, findings from the interview analysis, observations of system interaction, and the iterative refinements that followed each test cycle.

### Participant Characteristics

Fifteen female nurse assistants from 4 publicly operated (nonprivate) primary care centers representing urban, rural, and remote rural settings participated in the study (Table 1). All 15 nurse assistants participated in the interview data collection, while 14 participated in prototype testing and system interaction observations. Their ages ranged from 24 to 64 years (mean 47.2, SD 15.4 years; median 56, IQR 34-60 years), and their professional experience ranged from 5 to 43 years (mean 28.1, SD 15.6 years; median 39, IQR 16-41 years). All participants had previous experience with bladder scanning, while none had previous experience with AI-assisted health care technologies. The differences between the mean and median values reflected the distribution of the small sample, with several younger participants and participants with shorter professional experience lowering the respective means.

**Table 1:** Characteristics of participating primary care centers.

Primary care center	Setting	Nurse assistants, n	Registered patients, n
A	Rural	3	6500
B	Urban	6	11,000
C	Urban	4	10,500
D	Remote rural	2	3500

### Interview Findings

#### Overview

The interview analysis identified 4 themes describing nurse assistants’ perspectives on the AI-assisted carotid ultrasound system and its potential integration into preventive work within primary care. The themes capture key conditions influencing feasibility and implementation, as identified by participants, related to technology fit, system guidance, learning and confidence development, and the patient encounter.

#### Theme 1: Technology That Fits the Everyday Primary Care Work

The nurse assistants emphasized that the feasibility of the ultrasound technology depended on how well it aligned with the practical conditions of everyday primary care work. A central prerequisite for implementation was that the equipment fit within existing physical spaces, workflows, and time constraints, without introducing additional complexity or disruption.

Ergonomics and mobility were described as particularly important for routine use. The nurse assistants noted that examination rooms were often small and shared, increasing the need for compact equipment that was easy to position, adjust, and store. Stable working positions and flexible height adjustment were highlighted as important for minimizing physical strain during examinations.

Time pressure was also a recurring concern. The nurse assistants referred to tightly scheduled appointments and limited tolerance for technical delays, emphasizing that the system needed to be quick to initiate, reliable, and compatible with existing appointment structures. Provided that these conditions were met, they expressed optimism that the ultrasound examination could be incorporated into the VIP workflow.

#### Theme 2: System Guidance as a Prerequisite for Effective Use

The nurse assistants experienced the system as generally intuitive, yet aspects of the guidance and interaction design created uncertainty during the examination. They described difficulty attending to on-screen instructions while maintaining a stable probe position, which led to divided attention and disrupted workflow.

Clear and immediate feedback was therefore considered essential at key stages, including confirmation of correct anatomical positioning, readiness for image acquisition, and completion of recording. The nurse assistants also emphasized the need for a simplified interface to reduce cognitive load under time pressure and minimize errors when navigating similar options.

They highlighted the importance of concise, easily accessible instructions that could be followed in real time without shifting attention away from the patient or probe. Flexibility in guidance formats was seen as beneficial to support different learning preferences.

Time constraints further influenced usability. Some nurse assistants found the predefined recording time restrictive, contributing to stress when acceptable image quality was not achieved immediately. Overall, they emphasized that clear guidance and flexible interaction were critical for maintaining focus, confidence, and workflow continuity.

#### Theme 3: Practical, Peer-Supported Learning for Confidence in Clinical Use

The nurse assistants described the ultrasound examination as a practical skill primarily acquired through hands-on experience in clinical work. While introductory training provided a basic understanding, they emphasized that confidence developed through repeated use, including exposure to variation in anatomical conditions.

Peer-supported learning was described as particularly valuable. Opportunities to observe colleagues, ask questions in real time, and receive immediate feedback were seen as reducing uncertainty and supporting confidence before independent use. Practicing together prior to performing examinations on patients was considered an important step in the learning process.

The nurse assistants also highlighted the need for accessible learning resources that could be consulted when needed, as well as sufficient protected time for training prior to clinical implementation. This allowed the procedure to become familiar and less stressful to perform. Learning the new technology was further described as professionally rewarding, contributing to increased engagement in preventive care.

#### Theme 4: Confidence in the Patient Encounter

The nurse assistants described professional confidence in interactions with patients as essential for using the ultrasound technology in practice. Motivation to perform the examination was strengthened when it was perceived as contributing meaningful information for disease prevention.

Clear and supportive communication was emphasized as central to maintaining confidence during the examination. The nurse assistants highlighted the importance of patients understanding the purpose of the procedure, what was being assessed, and how results would be communicated. As nurse assistants were not responsible for interpreting the ultrasound images, their role in patient support was limited to providing information about the examination procedure. Questions concerning ultrasound findings, their clinical significance, or cardiovascular risk were to be referred to the licensed nurse or other responsible health care professional. Clear role boundaries and referral pathways were therefore considered important for supporting nurse assistants in the patient encounter.

They also emphasized the need to provide clear information to reduce patient uncertainty, including combining verbal explanations with written or other supportive materials. Overall, confidence in the patient encounter was closely linked to role clarity, trust in the broader care process, and clear communication structures.

### System Interaction Observations

System interaction observations were conducted with the 14 nurse assistants who tested the prototype. The observations revealed recurring usability challenges affecting ergonomics, probe handling, image acquisition, training, and system feedback during real-time scanning. These issues highlighted specific barriers to efficient task performance and workflow integration. A summary of the observed issues is presented in Table 2.

**Table 2:** Summary of observed usability issues during system interaction testing (n=14).

Theme and observed issues			Participants with observed issue, n
**Ergonomics and equipment positioning**			
	Screen placement was too far away or too high, requiring users to twist their bodies or reach during scanning	6	
	Lack of arm support made it difficult to initiate recordings, particularly when scanning the contralateral side	9	
	Differences in room layout and working positions affected ergonomic positioning of the equipment	—^a^	
**Probe handling**			
	The probe was held too far from the contact surface	11	
	The correct probe grip required coaching during the session	8	
	Probe orientation affected the ability to maintain the artery within the image and follow it toward the bifurcation	—^a^	
**Image acquisition**			
	Difficulty keeping the artery centered in the image, particularly near the carotid bifurcation	10	
	Difficulty following the artery toward the jawbone; guidance was required	8	
	Difficulty fully following the artery to the bifurcation before the recording time expired	5	
**Training gaps**			
	Procedural steps not consistently covered during the introductory instruction included gel application and patient neck positioning	11	
	Additional guidance was required during testing	10	
**System feedback**			
	Participants did not actively review the feedback screen when available	12	
	Scan approval did not always correspond to the observed image quality.	—^a^	

^a^A participant-level count was not applicable or was not systematically recorded for the observation.

The performance of the AI-based quality assessment was not formally evaluated in this study; observations related to scan approval should therefore be interpreted as qualitative usability observations rather than as a systematic assessment of model performance.

### Iterative Refinements

Findings from system interaction observations and interviews resulted in iterative refinements to both the prototype and the training approach throughout the test cycles. These refinements addressed identified barriers to real-world use and focused on improving ergonomics, usability, learning support, and clarity. The most significant updates are summarized in Table 3. Figures 5 and 6 illustrate the hardware and software components of the posttest prototype.

Overall, the iterative refinements addressed key usability barriers identified during testing and were intended to improve system stability, interaction clarity, and workflow integration in primary care settings.

**Table 3:** Iterative refinements to hardware, software, and training based on usability observations and interview feedback.

Component	Iterative refinements
Hardware	An additional cooling fan was installed to prevent overheating during extended use A mouse-activity device was added to prevent system freezes caused by inactivity A redesigned probe holder was introduced to ensure secure placement and reduce the risk of accidental dropping Planned refinements include an MDR^a^-certified touchscreen to meet medical hygiene standards, an adjustable screen arm to improve ergonomics, and a slimmer casing for the electronic components to reduce bulk
Software	Audio feedback was added to confirm the start and stop of measurements Data entry was simplified and partly automated, including replacing age input with the birth year and enabling automatic switching sides Interface clarity was improved through redesigned buttons and expanded screens, including enlargement of the start/stop button for easier activation An introductory data entry screen with reminders was added to guide users before starting measurements Additional safeguards were introduced, including prompts for missed measurements and loading screens to clarify system status Visualization options were added to allow different modes for displaying the segmentation mask Software bugs affecting navigation and usability were corrected Planned refinements include further development and validation of the AI-based image-quality assessment model to reduce the risk of incorrect quality approvals
Training materials and introductory instruction	Terminology was refined and explanatory images were added to clarify anatomical landmarks and scanning steps Training materials were updated with revised screenshots and summary slides for quick reference Greater emphasis was placed on key scanning steps, including following the artery to the mandibular angle and maintaining correct probe orientation Guidance on patient positioning was added to support ergonomics and examination consistency Additional instruction was provided on probe orientation to facilitate intuitive mapping between probe movement and image movement on the screen Training sessions were adapted to include live demonstrations and guided practice where needed

^a^MDR: medical device regulation.

**Figure 5:**
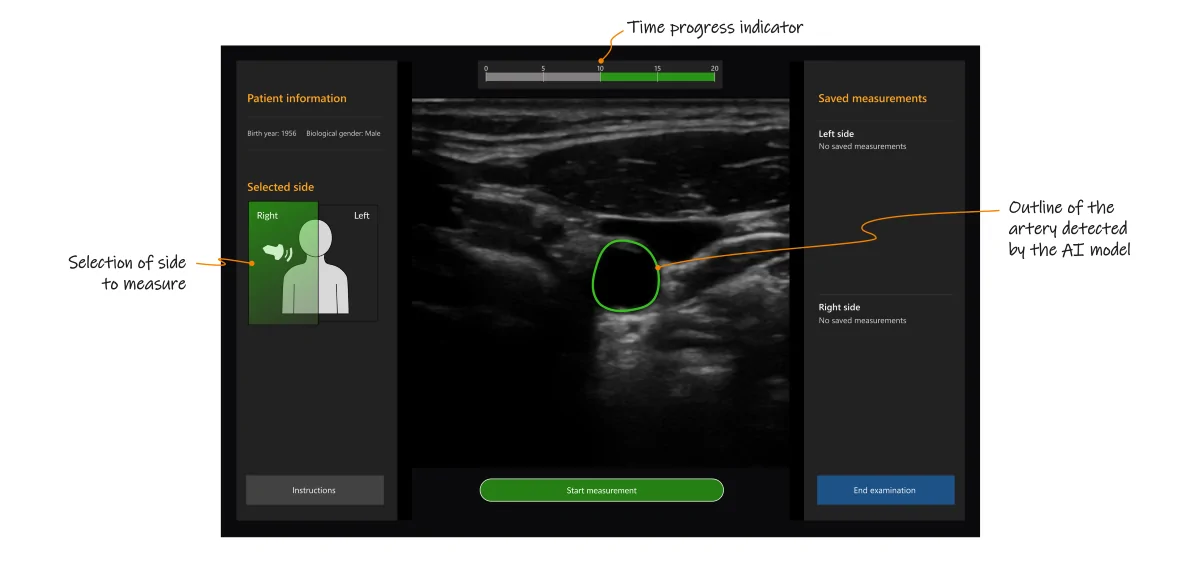
Screenshot of the posttest software.

**Figure 6:**
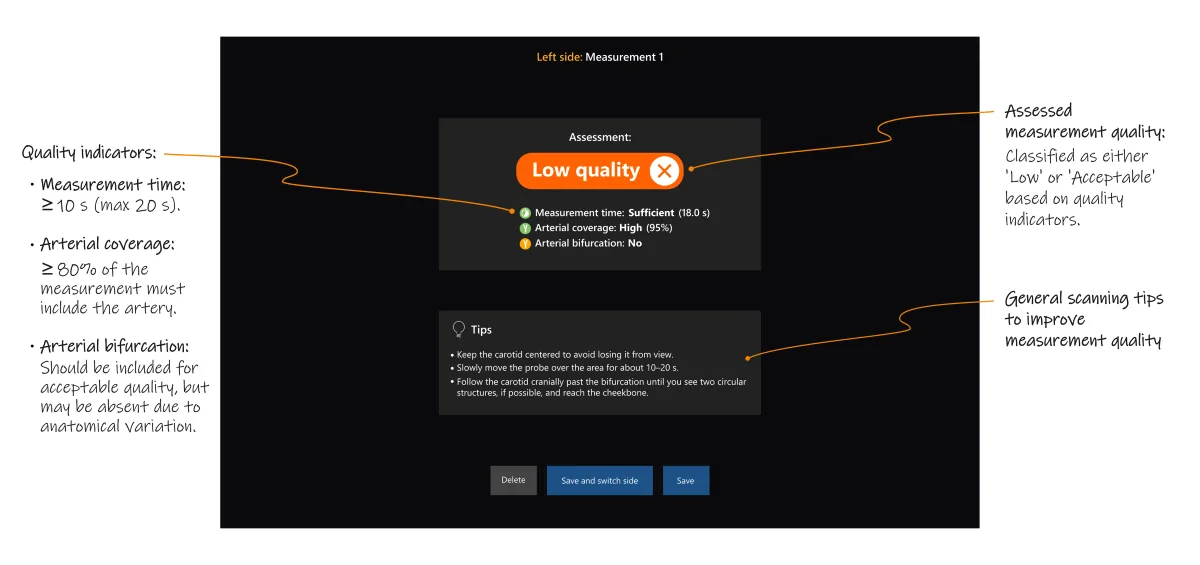
Screenshot of the posttest software (result screen).

## Discussion

### Principal Findings

This study describes an iterative user-centered design process for developing methods to support the use of AI-assisted carotid ultrasound by nurse assistants within routine preventive care in primary care settings. Findings from interviews and observational system interactions informed user-driven refinements to training approaches, hardware configuration, and software functionality, while also clarifying key usability requirements for integration into everyday clinical practice and the VIP. Participants expressed generally positive attitudes toward both the system and the surrounding methodology, including the opportunity to acquire a new technical skill and the potential to incorporate the examination into preventive care. At the same time, the findings highlight that successful integration depends on addressing practical conditions specific to the primary care context, including ergonomic and spatial constraints, time pressure and workflow demands, cognitive and interactional demands during the examination, training and support structures, and confidence in the patient encounter. Time constraints were a particularly prominent factor, influencing both usability during the examination and the perceived feasibility of integrating the method into routine primary care workflows. These conditions were reflected across 4 key areas: alignment with everyday workflow, the need for clear and supportive system guidance, the importance of practice-based and peer-supported learning, and confidence in patient communication.

Taken together, the iterative user-centered design process suggests that enabling nonexpert use of AI-assisted carotid ultrasound in primary care requires more than technical functionality alone. Instead, the system must combine ergonomic and spatial adaptability, intuitive interaction design, opportunities for practice, and support for communication in the patient encounter. Observational findings highlighted concrete usability and workflow challenges during real-time scanning, while interview findings provided insight into how these challenges were experienced and managed by nurse assistants, underscoring the importance of combining behavioral and experiential data in early-stage development. These findings extend previous research on AI-assisted ultrasound by illustrating that technical capability alone is insufficient for successful translation into routine care. The findings further suggest that AI support does not eliminate complexity but redistributes it, shifting demands from image interpretation to interaction with guidance systems, workflow coordination, and user attention during real-time scanning. Implementation therefore requires alignment between the technology, professional roles, and organizational conditions within everyday clinical practice [46,47].

Recent studies across several ultrasound applications support the potential of AI guidance to reduce operator dependence and enable nonexpert image acquisition. AI-assisted systems have enabled novices to acquire diagnostic-quality images in lung ultrasound and echocardiography, while similar benefits have been reported for vascular ultrasound applications, including carotid examinations and deep vein thrombosis assessment [11,48-50]. Comparable findings have also been reported in obstetric ultrasound, where AI-enabled smartphone-based systems allowed health care providers with varying levels of experience to perform scans of sufficient quality for AI or expert interpretation [51]. Together, these studies suggest that AI-assisted ultrasound may broaden access to imaging beyond specialist settings. However, they also highlight the continued importance of training, guidance, image quality feedback, and implementation support. While most previous studies have primarily focused on diagnostic performance and image acquisition, the present study extends this literature by examining the practical, organizational, and workflow-related requirements for integrating AI-assisted ultrasound into routine primary care practice.

### Iterative User-Centered Process

In this study, a tailored, in-context, and user-centered iterative approach was used to design system software, hardware, interface design, and training methods while exploring users’ perceptions of the system and its potential integration into everyday work within primary care and the VIP. User-centered development efforts are shaped by their target contexts but guided by shared principles, including early end user involvement, iterative refinement, and alignment with everyday clinical workflows and organizational structures [47,52].

In PRIMVIZA, the primary care context introduced additional complexity due to heterogeneity between units in terms of physical space, workflows, and professional roles. Consequently, both the emerging system and the research process required continuous local adaptation, including flexible training formats and varied interview arrangements to accommodate time constraints and clinical priorities. Rather than constituting methodological weaknesses, this flexibility enabled the development process to remain grounded in everyday primary care practice.

Insights from usability observations and interview feedback informed refinements to the prototype and associated training approach throughout the development process. These refinements addressed operational stability, interface clarity, ergonomics, workflow support, and training design, with the aim of reducing cognitive load and uncertainty during system use. Training methods were adapted in parallel with system development, with progressive refinement of terminology, visual aids, and training formats to accommodate users with limited ultrasound experience and local contextual constraints. Such changes reflect formative usability work, where early testing in realistic settings is used to refine functionality and workflows prior to large-scale deployment [53,54].

This aligns with implementation-oriented approaches that emphasize context-sensitive adaptation as a way to design for implementation rather than treating implementation as a downstream phase [47]. Accordingly, technical challenges, training needs, and contextual constraints encountered during the study were treated as inputs to redesign, contributing to a more practice-relevant system.

### Implementation Considerations

The interview findings indicate that the feasibility of introducing AI-assisted carotid ultrasound within the VIP depends both on technological characteristics and on how well the system aligns with the everyday organization of primary care work. Descriptions of space constraints, mobility needs, ergonomics, and time pressure highlight the importance of a good “fit” with local workflows as a central implementation condition. This aligns with implementation frameworks that emphasize compatibility and adaptability as key determinants of sustainable uptake [55,56]. From this perspective, the revisions described above can be understood as early adaptation work targeting physical, temporal, and social friction points that might otherwise hinder the normalization of the method within routine preventive care. Time constraints emerged as a central implementation factor, influencing both the feasibility of integrating the examination into existing workflows and usability during real-time scanning. In time-limited primary care settings, even small inefficiencies, uncertainties, or interruptions may have disproportionate effects on adoption and sustained use. Efficient workflow integration is therefore likely to be important for successful implementation.

Reliable image-quality assessment is another important consideration before clinical implementation. During prototype testing, scan approval did not always correspond to the observed image quality. However, the performance of the AI-based quality assessment was not formally evaluated in this study, and the frequency of discrepancies between scan approval and observed image quality was not systematically recorded. These observations should therefore not be interpreted as a systematic assessment of model performance. Acceptance of images of insufficient quality could affect subsequent image analysis and, consequently, the reliability of the visual information used in the preventive health dialogue. Further development and validation of the image-quality assessment model are therefore required before clinical implementation.

Another important consideration concerns the relational and professional dimensions of implementation. Preferences for clear system guidance, low cognitive load, predictable feedback, and peer-supported learning indicate that usability, professional confidence, and implementation are closely intertwined. In line with normalization process theory, successful integration will therefore require not only technical competence but also shared understanding, collective learning, and role clarity in the patient encounter [57]. This includes explicit clarification of professional responsibilities in relation to patient communication, particularly when visual outputs are generated by AI-assisted systems but interpreted and discussed by other members of the care team. In the proposed PRIMVIZA workflow, nurse assistants perform the ultrasound examination but do not interpret the findings; questions regarding the findings or their clinical implications should therefore be addressed by the licensed health care professional responsible for the subsequent preventive health dialogue. Clear role boundaries and communication pathways may therefore be essential to maintain trust, avoid misinterpretation, and support responsible risk communication in preventive settings [21].

Taken together, the findings suggest that future scale-up of the PRIMVIZA method will need to address technology, workflows, training arrangements, and patient communication in parallel, supported by the strong engagement and positive attitudes expressed by nurse assistants.

### Economic Considerations

The findings also provide preliminary insight into potential cost considerations associated with introducing AI-assisted carotid ultrasound within the VIP. In addition to expected costs such as equipment, training materials, and an initial demonstration session, the findings suggest that an additional setup cost may arise in the form of a learning period between training and the introduction of ultrasound in routine practice.

From an economic evaluation perspective, it would therefore be important to prospectively identify and measure nurse assistants’ time use associated with such a learning period. In the present study, approximately 2 hours per site were spent on demonstrations (including both participants and research staff, excluding travel time). Outside a research setting, less time would likely be required; however, the findings suggest that a face-to-face demonstration may represent a valuable investment to support effective system adoption.

These observations are in line with previous recommendations that highlight the importance of considering costs related to development and staff training when planning future scale-up of the AI-assisted carotid ultrasound system and its associated training approach [58].

### Generalizability and Implications

This study was conducted within the Swedish primary care context and the VIP, a structured cardiovascular prevention program with established routines for risk assessment and patient communication. The PRIMVIZA research described in this paper aims to assess feasibility, develop methods, and explore user perceptions through an end user, in-context approach, using VIP as a testbed for primary care–based CVD prevention. While VIP represents a long-established and well-developed preventive infrastructure [33], the principles and implementation conditions identified in this study may also be relevant beyond this specific context.

Similar structured health consultation models are currently being implemented across Swedish regions within the national structured health dialogue program, where CVD prevention constitutes a central focus area [59]. While organizational structures and preventive programs vary across health care systems, the core implementation challenges identified in this study, including time constraints, workflow integration, and support for nonexpert users, are likely to be relevant across settings introducing AI-assisted imaging technologies. These findings therefore suggest that, although the specific workflow developed within PRIMVIZA is context-dependent, the underlying requirements for successful integration may be transferable to other primary care environments.

From this perspective, the findings have broader implications for primary care prevention beyond the VIP context. The proposed approach to visualizing subclinical atherosclerosis may support more accessible, locally delivered, and person-centered preventive care, particularly in rural and remote settings [36]. However, successful implementation will also depend on organizational conditions that extend beyond the scope of the present study, including staffing levels, managerial support, and access to structured training and technical support at regional or system level. As with other task-shifting initiatives in primary care, introducing AI-assisted ultrasound may involve negotiation of professional boundaries and responsibilities, requiring organizational readiness and leadership engagement to support constructive implementation [5,47].

### Limitations

This study should be interpreted as a development study rather than an evaluation of clinical effectiveness. Demonstrating sustained clinical effects will require larger studies with longitudinal follow-up, comparative designs, and formal health economic evaluation. Nevertheless, such formative work is necessary to establish technical stability, usability, and implementation feasibility before larger trials can be meaningfully undertaken. The study was conducted using a prototype system under ongoing development, and the findings should therefore be interpreted in light of the evolving nature of both the technology and the associated workflow.

The work was conducted over a limited time period and in a small number of primary care units, within a context partly shaped by VIP-related routines and an established culture of structured preventive health dialogues. This may limit transferability to primary care settings where preventive consultations are less embedded in everyday practice or where organizational conditions, including staffing levels, physical space, scheduling constraints, and professional attitudes, differ. In addition, participating primary care units and nurse assistants actively chose to take part in the study and may therefore represent contexts and individuals with a more positive initial orientation toward innovation and new ways of working, which could have influenced perceptions of feasibility and usability. All participating nurse assistants were women, reflecting the gender distribution within the profession in the participating primary care centers. Nevertheless, perspectives from male nurse assistants were not represented in the study.

Furthermore, all prototype testing was conducted using a single, young, healthy standardized scanning participant. While this provided consistent anatomical conditions across sites and iterations, it limited the anatomical variability encountered during testing. Consequently, usability findings related to image acquisition, including probe handling and tracking of the carotid bifurcation, cannot be assumed to generalize to patients with more challenging anatomy, such as obesity, a short neck, or tortuous or atherosclerotic vessels. More broadly, the feasibility of the current workflow cannot be assumed to extend to real clinical patients and requires further validation in clinically representative populations. Future testing should therefore include participants with greater anatomical variability representative of the population in which the method is intended to be used.

As the research team was closely involved in the iterative development process, there is a potential risk of interpretive bias in observations and analyses. This is particularly relevant for the structured observations, as the observer (KJ) was also involved in the development and iterative refinement of the prototype, and her prior knowledge of the system may have influenced which usability issues were noticed and how observations were interpreted. To broaden the interpretation, observation findings and analytic decisions were discussed within the multidisciplinary research team, including researchers who were not directly involved in conducting the observations, and analytic decisions were documented throughout the process.

Participants were health care professionals with prior clinical experience, which may have influenced how they articulated usability, feasibility, and implementation-related considerations, as well as how these perspectives were interpreted in the analysis.

### Future Research

The findings of this study point to several important directions for future research and implementation. The first priority is to evaluate the technical and clinical validity of AI-assisted carotid ultrasound performed by nonexpert users. This should include testing the complete workflow in patients representative of the intended clinical population, including relevant anatomical and clinical variability, to assess its feasibility under routine primary care conditions. In addition, future studies should compare image quality and key measurement outcomes from examinations conducted by nurse assistants with those obtained by experienced biomedical analysts to assess equivalence and inform further refinement of the system and training methods. Formal validation of the AI-based image-quality assessment is also needed to determine how reliably the system distinguishes images of sufficient and insufficient quality before its use in clinical practice.

In parallel, future research should incorporate patient perspectives to better understand how these examinations and their visual outputs are experienced in a preventive care context. This includes exploring patients’ experiences of undergoing carotid ultrasound as part of routine VIP visits, as well as how visualization of subclinical atherosclerosis is perceived and used in preventive health dialogues. Such work will help clarify the communicative and motivational potential of visualization-based prevention and its role in supporting person-centered cardiovascular risk communication.

Beyond individual encounters, subsequent implementation studies should examine the feasibility of integrating AI-assisted carotid ultrasound as a routine component of the VIP in a limited number of primary care units. Initial testing should focus on the full VIP workflow, from ultrasound examinations performed by nurse assistants to nurse-led health dialogues, in a small number of health centers. This will be necessary to evaluate scalability, local adaptation needs, and resource implications, as well as to identify organizational and professional factors that support or hinder sustained use in everyday practice. In particular, future studies should further investigate how time constraints, workflow integration, and support for nonexpert users influence adoption and long-term use.

Finally, future development and evaluation of this approach should be situated within the broader landscape of AI-assisted prevention in primary care. Ensuring compatibility with parallel digital developments may support more efficient and targeted preventive practices, reduce manual workload, and facilitate integration within existing primary care services.

Taken together, these steps will enable progression from formative development toward broader implementation and evaluation, while maintaining a focus on usability, contextual fit, and preventive value. This staged approach is critical for ensuring that AI-assisted preventive technologies are introduced into primary care in a safe, acceptable, and sustainable manner.

### Implications for Future Technology Development

The findings of the present study may also have implications for the development of future AI-assisted ultrasound technologies. In particular, the identified needs for intuitive user guidance, ergonomic usability, workflow integration, and clear professional roles may remain relevant as ultrasound systems become increasingly miniaturized or wearable [60]. One example of such emerging technology is the “Sona” concept (Figure 7; [61]), a conceptual neck-worn ultrasound device for carotid imaging [61]. The concept was developed as part of a master’s project in collaboration with medical engineering researchers involved in PRIMVIZA, but was not developed or evaluated within the PRIMVIZA project. It is included as an illustrative example of how the user and implementation requirements identified in the present study may remain relevant to future technological approaches to carotid imaging.

**Figure 7:**
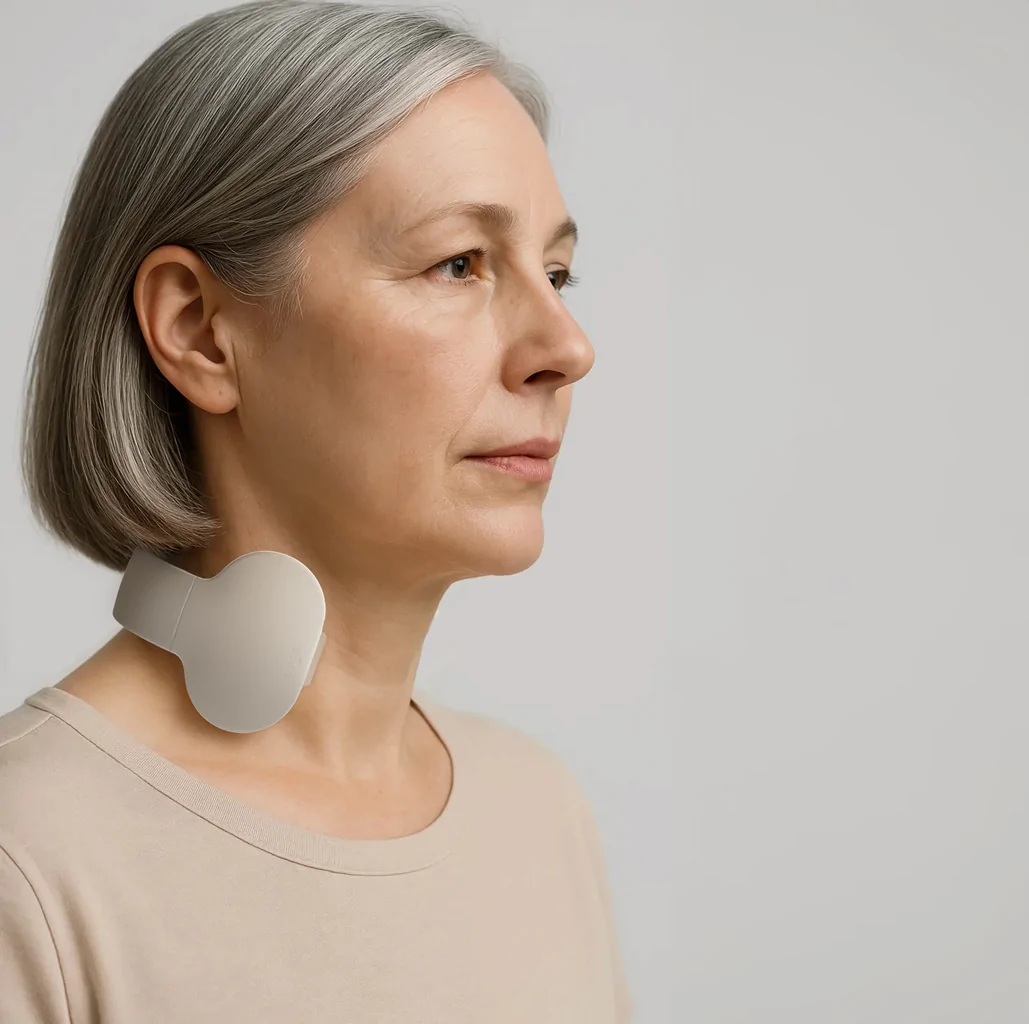
Conceptual design of a neck-worn ultrasound device (“Sona”) for carotid artery imaging. The image was generated using Sora (OpenAI).

### Conclusions

This user-centered study identified key practical, organizational, and educational conditions for enabling AI-assisted carotid ultrasound to be used by nonexpert staff in primary care. Rather than evaluating clinical performance, the study focused on understanding how such technology can be integrated into routine preventive practice and what conditions are required to support its use. Successful integration depends not only on technical capability but also on ergonomic fit, workflow compatibility, intuitive system guidance, opportunities for peer-supported learning, clear professional roles, and confidence in patient communication. These findings provide a foundation for future validation studies, patient-centered evaluations, and larger-scale implementation research.

## Data Availability

The datasets generated and analyzed during this study are not publicly available due to participant confidentiality and the risk of identification within the small number of participating primary care centers. Deidentified data may be available from the corresponding author on reasonable request, subject to applicable ethical and data protection requirements.

## Appendix

Multimedia Appendix 1 Technical description of the PRIMVIZA (Primary Care Prevention of Cardiovascular Disease Using Visualization of Subclinical Atherosclerosis for Improved Risk Communication) prototype.

Multimedia Appendix 2 Observation protocol.

Multimedia Appendix 3 Interview guide.

Multimedia Appendix 4 SRQR (Standards for Reporting Qualitative Research) checklist.

## Abbreviations

cIMT: carotid intima-media thickness
CVD: cardiovascular disease
PRIMVIZA: Primary Care Prevention of Cardiovascular Disease Using Visualization of Subclinical Atherosclerosis for Improved Risk Communication
SCORE2: Systematic Coronary Risk Evaluation 2
SRQR: Standards for Reporting Qualitative Research
VIP: Västerbotten Intervention Programme
VIPVIZA: Västerbotten Intervention Programme–Visualization of Asymptomatic Atherosclerotic Disease for Optimum Cardiovascular Prevention
