# Supplementary material for: AI-Assisted Carotid Ultrasound for Nonexpert Use in Primary Care Cardiovascular Prevention: User-Centered Iterative Development Study
Source: J Med Internet Res. 2026 Oct 8;28:e105250. doi: 10.2196/105250 (PMC13691457; doi:10.2196/105250)
Supplement: Supplementary file 1 [file jmir_v28i1e105250_app1.docx]

**Supplementary Appendix 1. Technical description of the PRIMVIZA prototype**

**AI model training procedure**

- The AI model was based on the YOLOv8 architecture for object detection and instance segmentation.
- The model was initialised using COCO-pretrained weights.
- Training was performed on n = 3,000 grayscale B-mode carotid ultrasound images obtained from the VIPVIZA study.
- Manually segmented carotid artery regions were used as ground-truth annotations.
- Training was conducted using a batch size of 16 and the stochastic gradient descent (SGD) optimiser.

**Image preprocessing pipeline**

- Ultrasound screen images captured from the ultrasound system were automatically cropped to include only the B-mode ultrasound image area.
- Images were resized to 256 × 256 pixels.
- Adaptive histogram equalization was applied (CLAHE).

**Ultrasound imaging preset**

The ultrasound system used the following imaging preset:

- B-mode depth: 4 cm
- Harmonic imaging
- Spatial compounding
- Flat time-gain compensation (TGC)

**Prototype hardware configuration**

The prototype consisted of the following hardware components:

- Touchscreen interface: Dell P2424HT (24")
- Processing unit: Lenovo P16v laptop running the AI software
- Ultrasound system: GE Vivid IQ Premium with L9 probe (GE Healthcare)
- Video capture device: Elgato Cam Link 4K

**Prototype dimensions**

The complete prototype had an approximate footprint of 59.5 × 54.5 cm (width × depth).
