# Supplementary material for: AI-Assisted Carotid Ultrasound for Nonexpert Use in Primary Care Cardiovascular Prevention: User-Centered Iterative Development Study
Source: J Med Internet Res. 2026 Oct 8;28:e105250. doi: 10.2196/105250 (PMC13691457; doi:10.2196/105250)
Supplement: Supplementary file 2 [file jmir_v28i1e105250_app2.docx]

**Patient position**: supine / sitting

| **Task** | **Assumption** | **P0X** | **P0Y** | **Notes** |
| --- | --- | --- | --- | --- |
| 1 | Starting a new examination and entering age and gender |  |  |  |
| 2 | Protecting the patient’s clothes with paper |  |  |  |
| 3 | Positioning the equipment within easy reach |  |  |  |
| 4 | Applying an appropriate amount of gel to the probe  (not directly on the patient’s neck) |  |  |  |
| 5 | Turning the patient’s head appropriately to the side  (not too wide angle) |  |  |  |
| 6 | Spreading the gel on the patient’s neck |  |  |  |
| 7 | Selecting the correct side of examination in the GUI |  |  |  |
| 8 | Holding the probe close to the contact surface  (pen grip or similar) |  |  |  |
| 9 | Orienting the probe so that the green marker points towards the patient’s larynx |  |  |  |
| 10 | Supporting the arm on the patient for better stability |  |  |  |
| 11 | Placing the probe at the correct start position  (just above the collarbone) |  |  |  |
| 12 | Applying enough pressure on the probe to avoid shadows |  |  |  |
| 13 | Starting the recording |  |  |  |
| 14 | Keeping the artery centered in the image |  |  |  |
| 15 | Following the artery towards the patient’s jawbone  (until after the bifurcation) |  |  |  |
| 16 | Obtaining at least one approved measurement within five attempts on each side |  |  |  |
| 17 | Getting at least one approved measurement in 5 attempts on each side |  |  |  |
| 18 | Other: |  |  |  |
