# Supplementary material for: AI-Assisted Carotid Ultrasound for Nonexpert Use in Primary Care Cardiovascular Prevention: User-Centered Iterative Development Study
Source: J Med Internet Res. 2026 Oct 8;28:e105250. doi: 10.2196/105250 (PMC13691457; doi:10.2196/105250)
Supplement: Supplementary file 3 [file jmir_v28i1e105250_app3.docx]

**Interview guide**

- Can you describe some challenges you have encountered when communicating health risks to patients?
- How do you perceive that patients usually react when needs for changes in their lifestyle habits are expressed, related to cardiovascular disease or other conditions, during the VIP health dialogue?
- Based on your experience, what types of communication or information tend to work well in helping patients change their lifestyle habits? What works less well? Please give examples.
- What factors among healthcare professionals or within the healthcare system, and what factors among patients, do you think facilitate or hinder patients in making lifestyle changes?
- What do you believe are the most important factors influencing patients’ motivation to change their lifestyle habits or to follow medical recommendations?
- How do you perceive that patients integrate information about their health risks into their everyday lives?
